# Supplementary material for: Estimating the Effectiveness of Shielding during Pregnancy against SARS-CoV-2 in New York City during the First Year of the COVID-19 Pandemic
Source: Viruses. 2022 Oct 30;14(11):2408. doi: 10.3390/v14112408 (PMC9697040; doi:10.3390/v14112408)
Supplement: Supplementary file 1 [file viruses-14-02408-s001.zip › viruses-1967279-supplementary.pdf]

Supplementary information

Table S1. A list of parameters, definitions and priors used in the Bayesian inference in the model of pregnant patients and general population.

Parameter category	Symbol	Definition	Prior	Units	Range
Pregnant patients	λ_τ	force of infection of pregnant patients; 1 / (average time to challenge by the virus for pregnant patients)	uniform (0,1)	week^{-1}	[0,1]
	τ	1 / (average time lag between virus detection and antibody detection)	gamma (4,3)	week^{-1}	[0,5]
	σ	1 / (average time lag between middle infection and past infection)	uniform (0,1)	week^{-1}	[0,1]
	β	antibody decaying rate in pregnant patients	uniform (0,1)	na	[0,1]
	y_{00}	proportion of people who are never exposed yet by April 20th, 2020	beta (8,2)	na	[0,1]
	k_{01}	tool parameter in the initial condition reparameterization	uniform (0,1)	na	[0,1]
	k_{11}	tool parameter in the initial condition reparameterization	uniform (0,1)	na	[0,1]
	k_{10}	tool parameter in the initial condition reparameterization	uniform (0,1)	na	[0,1]
General population	α	infection fatality ratio for general population	uniform (0,1)	na	[0,1]
	ω	antibody decaying rate in general population based on ELISA test [34].	uniform (0,1)	na	[0,1]

Table S2. Posterior estimates of parameters in general population.

Parameter(unit)	Definition	Median	2.5%	97.5%
α (-)	Infection fatality ratio among general population	0.0077	0.0067	0.0087
ω^{-1}(days)	1/antibody decaying rate among general population	209	152	333

Table S3. The effective sample size (n_{eff}) and the Gelman—Rubin($\hat{R}$) diagnostic for the four models.

Parameter	Model	n_{eff}	$\hat{R}$	Parameter	Model	n_{eff}	$\hat{R}$
τ	1	9902	1	k_{10}	1	9954	1
	2	7876	1		2	8854	1
	3	9516	1		3	11699	1
	4	8251	1		4	8544	1
σ	1	10448	1	k_{11}	1	13671	1
	2	8744	1		2	13344	1
	3	11928	1		3	13863	1
	4	8241	1		4	10818	1
β	1	9982	1	λ_{11}	1	10128	1

	2	8005	1	λ_{21}	2	9273	1
	3	9154	1	λ_{22}		8502	1
	4	7953	1	λ_{31}	3	10948	1
y_{00}	1	9330	1	λ_{32}		9698	1
	2	10350	1	λ_{33}		9549	1
	3	8152	1	λ_{41}	4	12655	1
	4	5675	1	λ_{42}		7391	1
k_{01}	1	19436	1	λ_{43}		7333	1
	2	19826	1	λ_{44}		7116	1
	3	19595	1				
	4	17016	1				

Table S4. Estimation of effectiveness of shielding from the four models.

Model	Estimation of effectiveness of shielding (95% CrI)
Model 1	53.4% (23.5%, 72.1%)
Model 2	52.0% (16.4%, 71.1%)
Model 3	48.4% (11.4%, 67.8%)
Model 4	47.3% (6.1%, 67.5%)

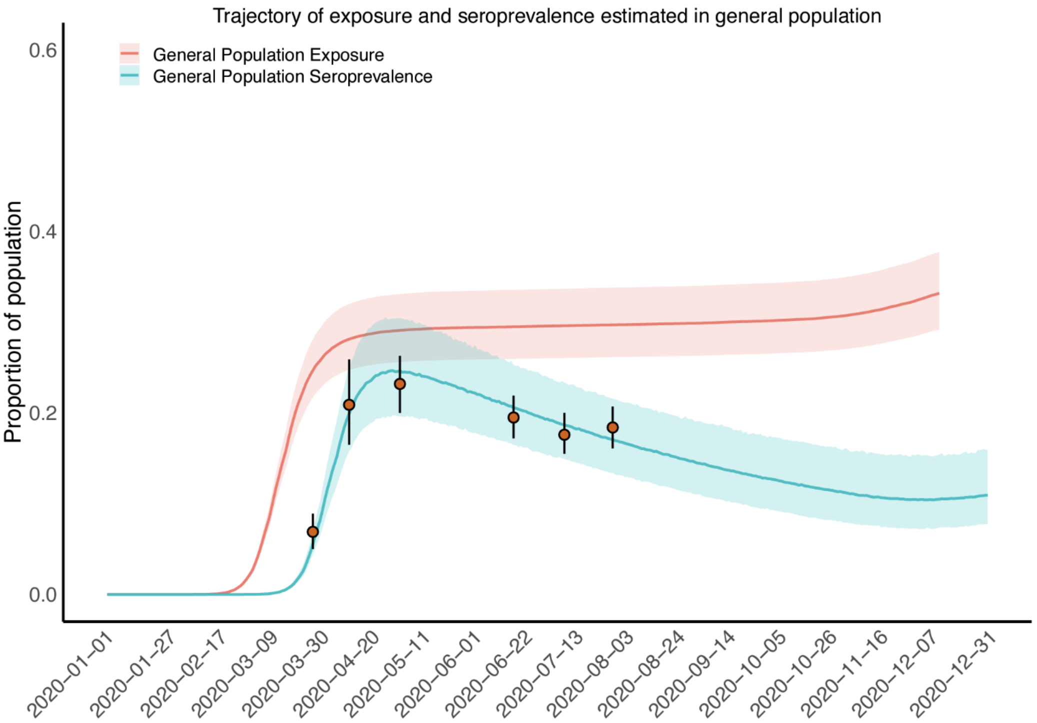

Figure S1. Time course of the SARS-CoV-2 infection among general from January 1st, 2020 to December 31st, 2020. The orange solid circles and black error bars represent the measured seroprevalence and their credible intervals respectively. The blue and orange lines show the median of predictions of seroprevalence and exposure among general population in New York City, while the shaded areas correspond to the 95% credible intervals.

We conducted a sensitivity analysis around the choice of prior of the initial conditional of proportion of pregnant patients who were not exposed previously by 20 April 2020 (numerically equals to 1 minus the level of exposure in pregnant patients by 20 April 2020). The results showed that the median and 50% credible band of posterior estimates are very robust (**Error! Reference source not found.**) although a heavy left tail in the 90% and 95% credible band (**Error! Reference source not found.**) are estimated when the priors are very weak, for example

uniform (0,1). However, considering the transmission speed and antibody decaying rate it is reasonable to choose a relative formative prior, such as beta (2,1) and beta (8,2) and then the posterior estimates are more concentrated around 0.85.

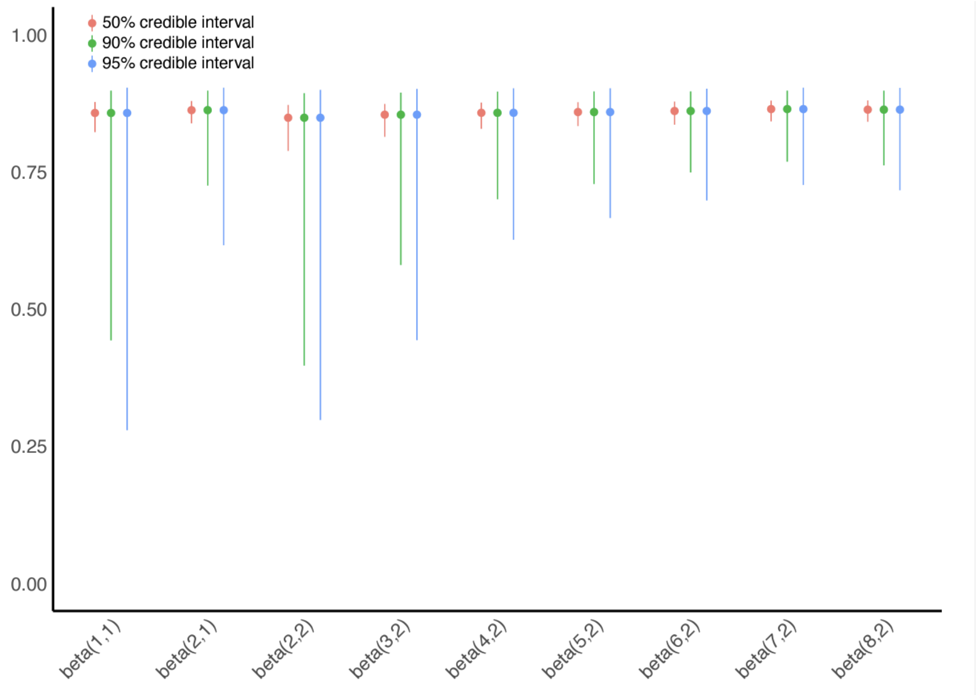

Figure S2. Comparisons of posteriors with different significant levels (50%, 90% and 95%) for the proportion of pregnant patients who were not exposed previously by 20 April 2020 (numerically equals to 1 minus the level of exposure in pregnant patients by 20 April 2020).

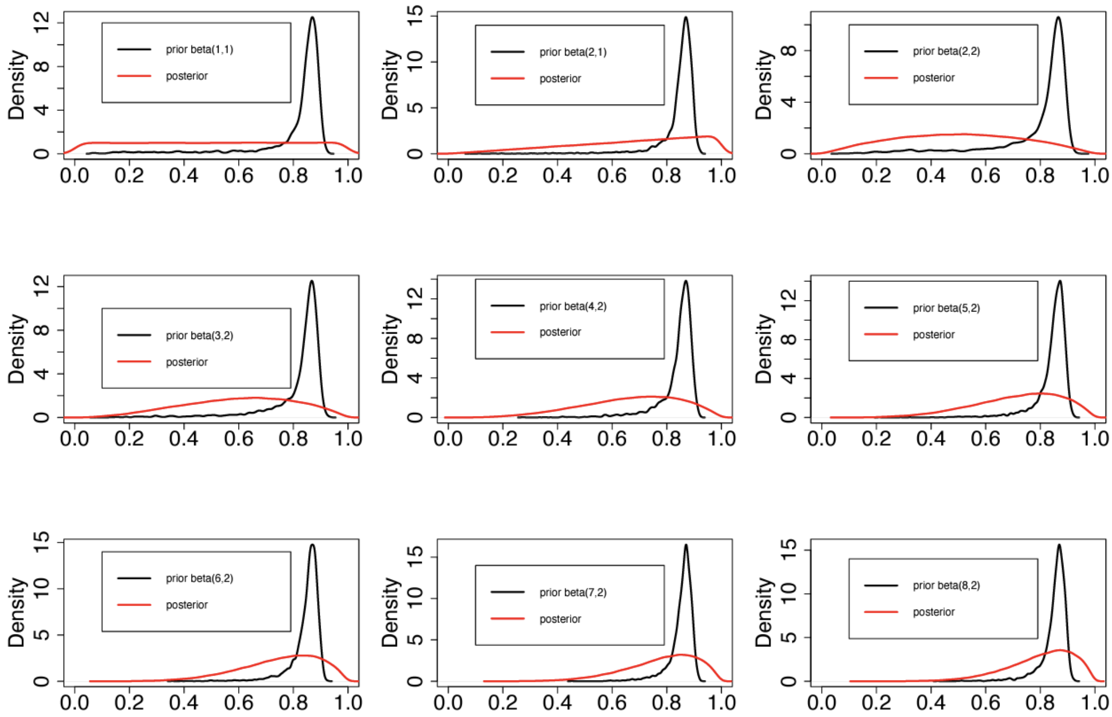

Figure S3. Comparisons of priors and posteriors for the proportion of pregnant patients who were not exposed previously by 20 April 2020 (numerically equals to 1 minus the level of exposure in pregnant patients by 20 April 2020).

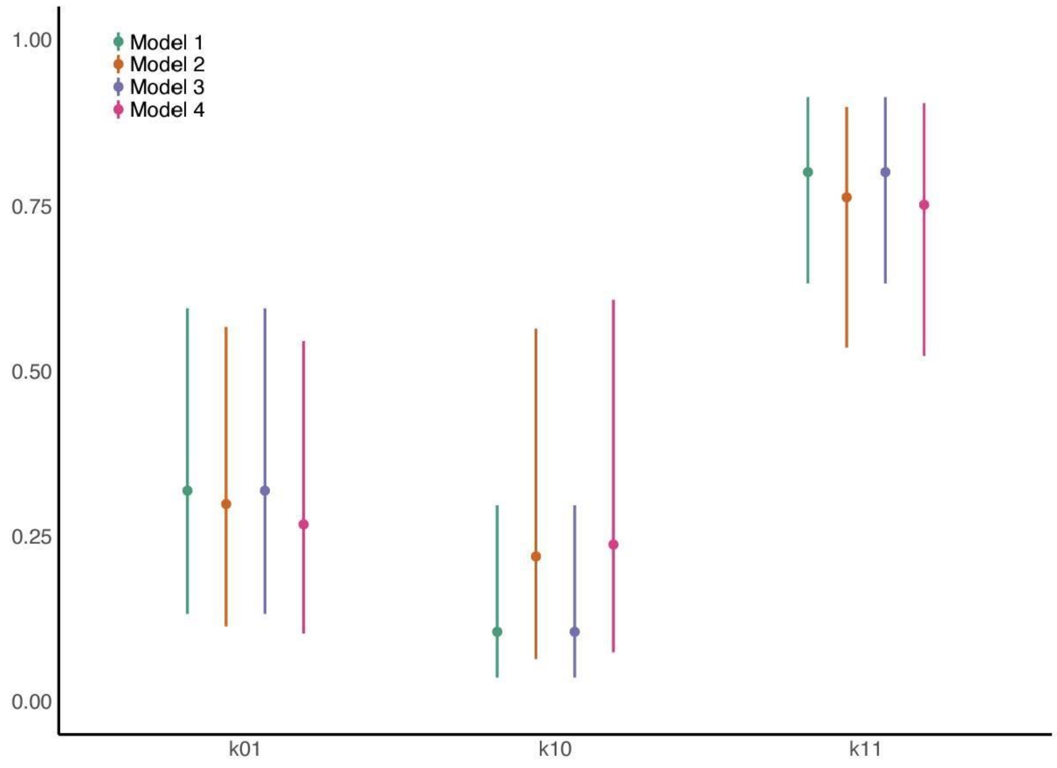

Figure S4. Comparison of estimates of 'instrumental parameters' among the four models.
